# Chemical Constituents of *Smilax china* L. Stems and Their Inhibitory Activities against Glycation, Aldose Reductase, α-Glucosidase, and Lipase

**DOI:** 10.3390/molecules22030451

**Published:** 2017-03-11

**Authors:** Hee Eun Lee, Jin Ah Kim, Wan Kyunn Whang

**Affiliations:** Pharmaceutical Botany Laboratory, College of Pharmacy, Chung-Ang University, Heukseok-dong, Dongjak-gu, Seoul 156-756, Korea; y-u-h-r214@hanmail.net (H.E.L.); jinakim@cau.ac.kr (J.A.K.)

**Keywords:** *Smilax china* L., bioactivity-guided isolation, advanced glycation end products formation inhibitory assay, aldose reductase inhibitory assay, α-glucosidase inhibitory assay, lipase inhibitory assay

## Abstract

The search for natural inhibitors with anti-diabetes properties has gained increasing attention. Among four selected Smilacaceae family plants, *Smilax china* L. stems (SCS) showed significant in vitro anti-glycation and rat lens aldose reductase inhibitory activities. Bioactivity-guided isolation was performed with SCS and four solvent fractions were obtained, which in turn yielded 10 compounds, including one phenolic acid, three chlorogenic acids, four flavonoids, one stilbene, and one phenylpropanoid glycoside; their structures were elucidated using nuclear magnetic resonance and mass spectrometry. All solvent fractions, isolated compounds, and stem extracts from plants sourced from six different provinces of South Korea were next tested for their inhibitory effects against advanced glycation end products, as well as aldose reductase. α-Glucosidase, and lipase assays were also performed on the fractions and compounds. Since compounds **3**, **4**, **6**, and **8** appeared to be the superior inhibitors among the tested compounds, a comparative study was performed via high-performance liquid chromatography with photodiode array detection using a self-developed analysis method to confirm the relationship between the quantity and bioactivity of the compounds in each extract. The findings of this study demonstrate the potent therapeutic efficacy of SCS and its potential use as a cost-effective natural alternative medicine against type 2 diabetes and its complications.

## 1. Introduction

Type 2 diabetes mellitus (T2DM), a disease caused by insulin resistance, currently represents a major health issue concerning both the governments of countries where patients live as well as affected individuals. Approximately 415 million people worldwide suffer from T2DM and the number is forecast to rise to 642 million by 2040 [1]. According to the World Health Organization (WHO), long-term uncontrolled diabetes can affect the functions of other organs, resulting in a series of complications, such as retinopathy, cataracts, neuropathy, atherosclerosis, nephropathy, and delayed wound healing [2]. In addition, persistent hyperglycemia causes the formation of advanced glycation end products (AGEs) via non-enzymatic glycation of amino acid residues and oxidative derivatives [3]. This elevates polyol and hexosamine pathway flux and boosts the activation of kinase C isoforms, which are considered the main factors in the pathogenesis of long-term diabetic complications [4]. Growing evidence has shown that accumulation of AGEs leads to irrevocable functional and structural modifications in proteins, like collagen, elastin, and albumin [5]. In this situation, when AGEs bind to AGEs receptor (RAGE), reactive oxygen species (ROS) are released and their downstream signaling basically results in induction of pro-inflammatory cytokines [6]. As a result, AGEs and the AGE-RAGE axis have thus been implicated in the pathogenesis of diabetic complications [7]. In addition, in the polyol pathway, aldose reductase (AR) catalyzes NADH-dependent reduction of glucose to the corresponding sugar alcohol, sorbitol, [8] which is an osmotically active alcohol that causes oxidative stress and leads to terrible tissue injuries [9], especially cataracts. Moreover, sorbitol and its metabolites accumulate in the retina, kidneys, and lens due to their poor efficiency of metabolism and short penetration across membranes, further resulting in diabetic complication development [10]. Therefore, AR and AGEs inhibitors are potential therapeutic agents for the treatment of diabetes and its pathogenic complications [11]. In addition, α-glucosidase inhibitors are selected as first-line drugs to prevent the absorption of carbohydrates after food intake [12]. Many recent studies have suggested that free fatty acids, which are formed during steatolysis by lipase, play a role in the development of diabetes [13,14]. Some commonly used synthetic anti-diabetic drugs, such as aminoguanidine (AMG), tetramethyleneglutaric acid (TMG), acarbose, and orlistat are known to have numerous side effects, including flatulence, abdominal pain, hepatic injury, renal tumors, acute hepatitis, abdominal fullness, and diarrhea [15,16,17,18]. Thus, medical plants, which are known to be safe, could represent a complementary and alternative option for the prevention and treatment of diabetes-related complications [19].

*Smilax china* L., a member of the Smilacaceae family, is widely distributed worldwide in tropical and temperate regions, especially in East Asia [20,21]. This plant is a perennial and somewhat woody climber with aculeated skin and paired tendrils that aid in climbing. Several studies have shown that the tubers of *Smilax china* L. have been used in traditional medicine for the treatment of furunculosis, gout, tumors, and inflammation [22,23,24,25,26]. Recently, there have been many studies discussing the use of *Smilax china* L. leaves. These studies reported that they have antioxidant, antimicrobial [21], antidiabetic [27], and anti-hyperuricemia effects [28] owing to the presence of significant amounts of polyphenols [29] such as rutin, kaempferin, and kaempferitrin [30]. However, the stems of *Smilax china* L., including its thorny vines that affect the growth of other plants, are usually discarded as a waste. Although there are very few studies focusing on the experimental use of *Smilax china* L. stem (SCS), it has been reported to show significant inhibitory activity against AGEs formation among 156 Korean herbal medicines [31]. In a previous study in our lab, we found that the SCS extract has potential therapeutic or preventive effects against obesity, hyperlipidemia, and fatty liver [32]. In both studies, the stem extract showed much stronger inhibitory activities that leaves extract did. Another study showed that the electron-donating ability and xanthine oxidase inhibitory activity of the SCS extract are more significant than those of the root extract [33], and the results imply that SCS has momentous antidiabetic potential because a strong relationship between antioxidant and antidiabetic activity has been reported in phytomedicine [34].

The aim of this study was to isolate and elucidate the structures of bioactive compounds from SCS and to investigate their inhibitory effects on AGEs formation, AR, α-glucosidase, and lipase enzyme activities to evaluate whether this stem can be used for the treatment of diabetes mellitus. In addition, we performed a quantitative study of the most active major compounds and showed the correlation between their contents and activity rates. This is the first report on investigating the effect of AR, α-glucosidase, and lipase enzyme activity potential from SCS.

## 2. Results and Discussion

### 2.1. Structural Identification of Isolated Compounds from SCS

Our phytochemical study of SCS was able to isolate and identify ten compounds. The spectral properties, NMR and MS, of these isolated compounds were found to be identical with those in previous studies. The purity of the obtained compounds, as measured by high performance liquid chromatography photo diode array (HPLC-PDA), was higher than 95.0%. Compounds **1**, and **6**–**10** were obtained from the ethyl acetate fraction (EAF) and compounds **2**–**5** were found in the *n*-butanol fraction (NBF).

Compound **1** was determined to be protocatechuic acid, according to the supporting information [35]. Compounds **2**, **3**, and **4** were identified as 5-*O*-caffeoylquinic, 3-*O*-caffeoylquinic, and 4-*O*-caffeoylquinic acids, respectively. Previous literature supported these conclusions [35,36,37,38,39]. The confusion between compounds **2** and **3** was resolved via a complete structural assignment using Heteronuclear Multiple Bond Correlation (HMBC) measurements. Compound **5** was characterized as kaempferol 3-*O*-β-d-glucopyranosyl-7-*O*-α-l-rhamnopyranoside. This conclusion was supported by previous literatures [40,41,42]. Compound **6** was characterized as quercetin 3-*O*-α-l-rhamnoside, with support from previous literature [43]. Compound **7** was characterized as kaempferol 3-*O*-α-l-rhamnoside, and this was supported by data from the literature [43,44]. Compound **8** was characterized as 3,5,4′-trihydroxy-*trans*-stilbene, by comparing the obtained spectroscopic data with previously published data [45,46]. Compound **9** was determined to be 2-feruloyl-*O*-α-d-glucopyranoyl-(1′→2)-3,6-*O*-feruloyl-β-d-fructofuranoside, by comparing these data with previous reports [47,48,49]. Finally, compound **10** was identified as isoscutellarein 8-*O*-rhamnoside, which was confirmed by comparing the reported values with those of a previous study [50]. Consequently, three chlorogenic acid derivatives (compounds **2**–**4**), one simple phenol (compound **1**), four flavonoids (compounds **5**–**7**/**10**), one stilbene (compound **8**), and one phenylpropanoid glycoside (compound **9**) were obtained from the 50% ethanol extract of SCS. Despite the significant therapeutic potential of SCS, there are few studies related to it and only one isolation study. In the previous study, nine phenylpropanoids, smilasides A–F, smiglaside E, helonioside B and 2′,6′-diacetyl-3,6-diferuloylsucrose were isolated from the chloroform layer of SCS [51]. In this study, however, ethyl acetate and *n*-butanol fractions were explored for isolation to the bioassay results. To the best of the authors’ knowledge, this is the first report of compounds **4**, **5**, and **10** being isolated from *Smilax china* L.

### 2.2. Bioactivity Assay

#### 2.2.1. Inhibition of AGEs Formation and AR Activity

##### Inhibition of AGEs Formation and AR Activity by Four *Smilax* Samples

In order to evaluate the inhibitory effect of SCS against AGEs formation and AR activity, 70% MeOH stem extracts of four common Smilacaceae family plants: *Smilax china* L., *Smilax sieboldii, Smilax riparia* var. ussuriensis, and *Smilax ferox* Wall. ex Kunth, were examined. All selected species showed promising and concentration-dependent inhibitory activities in both the AGEs and AR inhibition assays. However, among the four species, *Smilax china* L. was the most potent inhibitor in the AGEs and AR assays (Table 1). *Smilax ferox* Wall. ex Kunth showed the second strongest inhibitory activity. Since the SCS extract was the most potent inhibitor among the tested samples, it was selected for further investigation to elucidate the compounds active against AGEs formation and AR activity.

##### Inhibition of AGEs Formation and AR Activity by SCS Extract and Fractions

As previously mentioned, a 50% ethanol extract residue of SCS was partitioned with hexane, ethyl acetate, *n*-butanol, and water, yielding four different fractions. These four fractions, as well as the crude extract, were separately examined to determine their effect on AGEs formation and AR activity. The ethyl acetate fraction (EAF) showed the strongest inhibition of AGEs formation and AR activity (Table 2). In particular, its IC_50_ for inhibition of AGEs formation was even lower than that of the reference drug AMG. The *n*-butanol fraction (NBF) also showed potent inhibition of AGEs formation and AR activity compared to the positive controls AMG and TMG. In contrast, the hexane and water fractions possessed very weak inhibitory activities, demonstrating less than 50% inhibition at the tested concentrations. This indicated that EAF and NBF contained the active components of the SCS extract, and therefore, the components of both fractions were isolated via repeated open column chromatography for further examination.

##### Inhibition of AGEs Formation and AR Activity by EAF/NBF-Isolated Compounds

EAF/NBF-isolated compounds were examined using the same methodology used for the extracts and fractions. All tested samples showed concentration-dependent activities, as well as generally consistent inhibitory effects in both the AGEs formation and AR activity assays (Table 3). All tested compounds possessed lower IC_50_ values than the positive control (AMG) under assay conditions. Quercitrin (**6**), which is formed by glycosylation of quercetin at C-3, appeared to be the most potent component in the AGEs formation and AR activity assays compared to the reference drugs AMG and TMG. This result concurred with that of a previous structure activity relationship (SAR) study that indicated that AGEs formation inhibitory activity increased according to the number and position of hydroxyl groups (3′-, 4′-, 5-, and 7). Furthermore, this study showed that flavonols possessing the 7-hydroxyl and/or catechol moieties in the B ring exhibited strong inhibitory activity in the AR assay. Therefore, quercetin-type flavonols expressed higher inhibitory capacity in both bioassays than kaempferol-type flavonols, because quercetin-type compounds contain hydroxyl groups on those exact four positions, which means having 7-hydroxyl and catechol moieties on the B-ring, explaining why compound **7** exhibited weaker activities than compound **6** in both bioassays. In particular, *O*-glycosylation at the C-7 position of flavonols decreases AGEs formation inhibitory capacity [52], This explains the low inhibitory capacity of compound **5**, which was glycosylated at both the C-3 and C-7 positions. In addition, compound **10** exhibited weaker inhibitory activity against AGEs than compound **7**, but a stronger effect against AR. It can be suggested that 8-*O*-glycosylation tended to increase AR inhibitory activity. The inhibitory capacity of the stilbene **8** was weaker than that of the some flavonoid compounds, but still stronger than compounds **5** and **7** [53]. The three caffeoylquinic acids showed similar inhibition potentials. 3-*O*-Caffeoylquinic acid was the second most potent AR activity inhibitor. However, it was the fourth in terms of AGEs formation inhibition. In contrast, 4-*O*-caffeoylquinic acid was the second most potent compound in AGEs formation inhibition but the fourth in terms of AR activity inhibition. 5-*O*-Caffeoylquinic acid showed weaker inhibitory activity than the other two compounds in both assays. Finally, compounds **9** and **1** exhibited poor inhibitory activities compared to the flavonoids and chlorogenic acids. This finding suggests that compounds **3**, **4**, **6**, and **8** were the main factors affecting the SCS extracts’ potential against AGEs and AR inhibitory activities, and especially compounds **3** and **4** were responsible for the significant activities of NBF while compounds **6** and **8** plays an important role in EAF. We also found out that some pure compounds showed weaker activity than the extract or fractions, which means the potency of the extract was due to those four very potent compounds.

##### Inhibition of AGEs Formation and AR Activity by SCS Extracts from Six Different Regions of Korea

**Figure 1:**
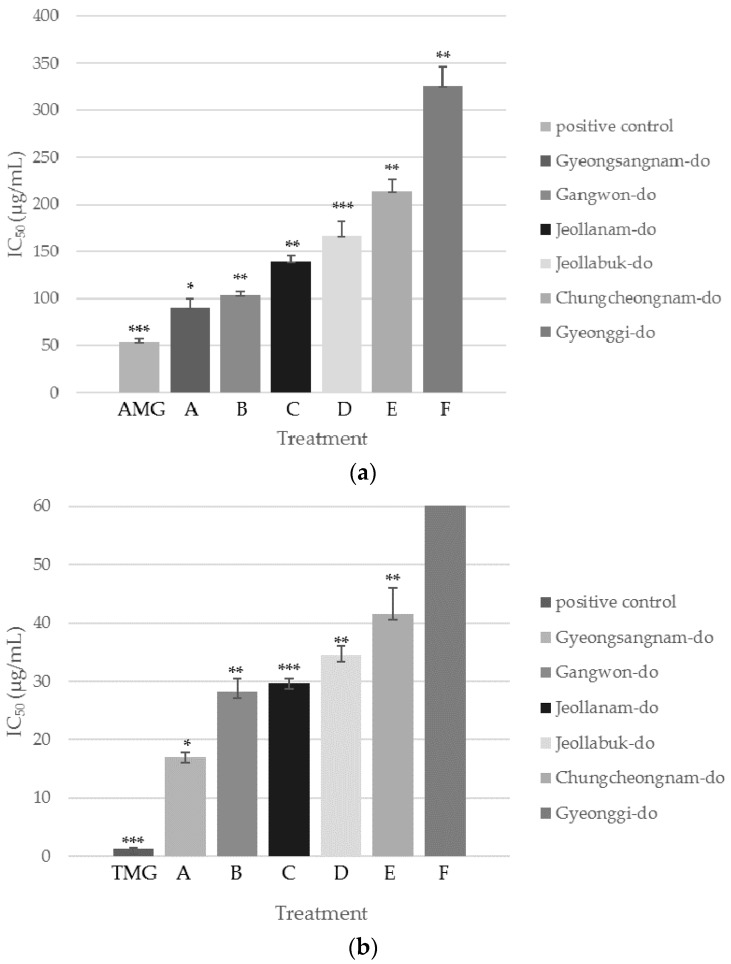
(**a**) IC_50_ values of SCS extracts from different provinces of Korea in AGEs assay; (**b**) IC_50_ values of SCS extracts from different provinces of Korea in AR assay. Values are presented as mean ± SD (*n* = 3). * *p* < 0.05 compared with the positive control group; ** *p* < 0.01 compared with the positive control group; *** *p* < 0.001 compared with the positive control group (Student’s *t* test).

Six samples of SCS were collected from different Korean provinces: Gyeongsangnam-do (sample A), Gangwon-do (sample B), Jeollanam-do (sample C), Jeollabuk-do (sample D), Chungcheongnam-do (sample E), and Gyeonggi-do (sample F). The inhibitory capacity of each sample against AGEs formation and AR activity increased in a concentration-dependent manner (Figure 1). Interestingly, the inhibitory activity ranking of these six samples was identical for both assays. Sample A showed the highest inhibition with IC_50_ values of 90.4 ± 9.4 µg/mL and 17.0 ± 0.9 µg/mL in the AGEs formation and AR activity assays, respectively. Sample B followed with IC_50_ values of 103 ± 4 µg/mL and 28.2 ± 2.3 µg/mL, then sample C with IC_50_ values of 139 ± 6 µg/mL and 29.7 ± 0.7 µg/mL, sample D with IC_50_ values of 167 ± 15 µg/mL and 34.4 ± 1.7 µg/mL, sample E with IC_50_ values of 214 ± 13 µg/mL and 41.5 ± 4.6 µg/mL, and finally, sample F showed the weakest inhibitory activity with IC_50_ values of 326 ± 20 µg/mL and 98.8 ± 6.0 µg/mL.

##### Quantitative HPLC Analysis of Four Bioactive Compounds from SCS Extracts

**Figure 2:**
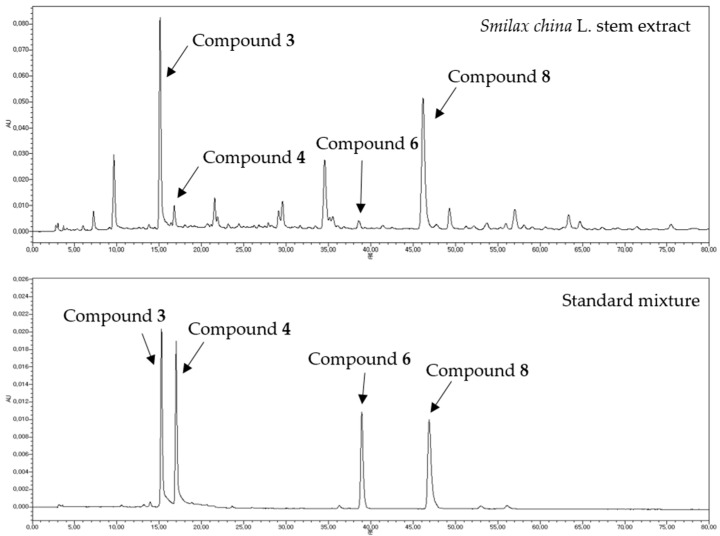
HPLC chromatograms of SCS 70% MeOH extract and four standard mixtures. Compound **3**: 3-*O*-chlorogenic acid (2.2.1.5.: 15.479); Compound **4**: 4-*O*-chlorogenic acid (Rt: 17.416); Compound **6**: quercitrin (Rt: 38.645); Compound **8**: *trans*-resveratrol (Rt: 45.640); HPLC conditions are described in the Section 3.7.

HPLC analysis was conducted for quantitative evaluation of SCS extract active components (Figure 2). After a preliminary screening of the collected samples, compounds **3** and **8** were identified as the major components of SCS extracts, with compound **3** being the most abundant in each of the samples and compound **8** ranking the second. The four compounds **3**, **4**, **6**, and **8**, which showed the most potent inhibitory activity against AGEs formation and AR activity, were examined. Extract components comprising 1.0 g of extract powder were quantified in µg (Table 4). The quantitative study consequently suggested that the IC_50_ values of the tested samples were inversely correlated with the total contents of the compounds **3**, **4**, **6**, and **8**. This indicated that the four examined compounds may play crucial roles in the inhibition of AGEs formation and AR activity. Indeed, sample A, the most potent SCS extract, possessed the highest amounts of these four compounds, and sample F, the least potent, possessed the lowest amount. This finding suggested that both those four compounds and HPLC analysis method can be used for quality control standards of SCS if this is used for medicinal purpose.

#### 2.2.2. α-Glucosidase and Lipase Inhibition Assays

##### Inhibitory Effect of SCS Extract and Its Fractions on α-Glucosidase and Lipase Activities

α-Glucosidase inhibitors are the most widely used agents for the treatment of diabetes mellitus. They act by preventing the digestion of carbohydrates [54]. Pancreatic lipase plays an important role in the digestion and absorption of triacylglycerols in the intestine, and dietary fat is not directly absorbed unless it has been hydrolyzed by lipase. Therefore, the inhibition of both these enzymes is important when examining the anti-diabetic and anti-obesity properties of natural plant materials. SCS extract, hexane-soluble fraction, EAF, NBF, and water-soluble fraction were examined for their α-glucosidase and lipase inhibitory activities. All samples demonstrated concentration-dependent inhibitory activities (Table 3). The EAF showed significant inhibitory activity against both enzymes. It was followed by NBF. The IC_50_ values of the EAF and NBF were roughly 7-fold and 2-fold lower than that of the well-known inhibitors acarbose, respectively. The hexane fraction also showed a strong lipase inhibitory activity. 

##### Inhibitory Effect of EAF/NBF-Isolated Compounds against α-Glucosidase and Lipase Activities

The α-glucosidase and lipase inhibitory activities of the previously isolated 10 compounds were evaluated (Table 4). The results showed that compound **8** possessed the most potent activity, and the two flavonoids, compounds **7** and **10,** appeared to be the second most potent α-glucosidase inhibitors, followed by compounds **9** and **6**. A previous study suggested that hydroxyl groups, especially at the C-5 and C-7 positions of the A–C ring and C-3′ and C-4′ positions of the B ring, increased the inhibitory activity of flavonoids against α-glucosidase [55,56]. However, glycosylation of flavonoids weakened its inhibitory effects on α-glucosidase owing to increasing molecular size and polarity, which lead to nonplanar structure. In this study, compound **6** showed weaker inhibitory activity than compound **7**, despite of compound **6** contained more hydroxyl group on B-ring. This result gave an evidence that quercetin is affected more by glycosylation than kaempferol. In addition, in previous study revealed that non-glycosylated stilbenes displayed about five fold higher inhibitory activity than glycosylated counterparts [57]. This is also because of steric hindrance caused by glycosylation, accordingly, strong activity of flat structure compound **8** provides the basis for a SAR. The remaining five compounds illustrated moderate inhibitory activity. The three caffeoylquinic acid-type compounds, compounds **2**–**4**, were known to inhibit α-glucosidases in a non-competitive manner [58]. In this study, we found out that among the three, 5-*O*-caffeoylquinic acid (compound **2**) is the strongest followed by 3-*O*-caffeoylquinic acid. 

As for the lipase inhibition assay, compound **1** showed the most prominent inhibitory activity against the lipase enzyme. Indeed, a previous screen of phenolic acids showed that the inhibitory effect of *ortho*-position substitution was stronger than that of the *para*-position. [59]. Compound **8** was the second strongest compound, followed by compounds **6**, **10**, **2**, and **7**. Many articles have indicated that *trans*-resveratrol (compound **8**) presented anti-obesity activity in both in vitro and in vivo models [60,61,62]. In addition, another study indicated that the presence of a hydroxyl moiety at the C-3 position of flavonoids enhanced pancreatic lipase inhibition [63]. However, unlike for α-glucosidase, lipase inhibition by a wide range of isolated flavonoids has not been studied (except for the catechin derivatives). Therefore, it is difficult to perform a SAR study. The remaining compounds exhibited mild lipase inhibitory activities. However, we still could still conclude that compounds **1**, **6**, **8**, and **10** were the important factors in the EAF responsible for lipase inhibition, and that compound **2** was the important factor present in the NBF.

## 3. Materials and Methods

### 3.1. General Information

The thin layer chromatography (TLC) plates used for analytical TLC in this study were Kieselgel 60 F_254_ (Merck, Darmstadt, Germany). The purity of the standard compounds used was more than 95.0% as determined by high performance liquid chromatography (HPLC). HPLC-grade acetonitrile, methanol, and water were purchased from J.T. Baker (Phillipsburg, PA, USA). HPLC-grade phosphoric acid (≥85 wt % in H_2_O) was obtained from Sigma-Aldrich Co. (St. Louis, MO, USA). Polyvinylidene fluoride (PVDF) membrane filters (13 mm, 0.45 µm) were purchased from Whatman (Maidstone, UK). Analytical balance AT460 was purchased from Mettler-Toledo (Columbus, OH, USA). The model Vortex Genie-2 vortex mixer was purchased from Scientific Industries (New York, NY, USA). The DH D300H ultrasonic cleaner was purchased from Daihan Scientific (Seoul, Korea) and the MF 550 centrifuge was purchased from Hanil Science Industrial (Gangwon, Korea). Diaion HP-20, Sephadex LH-20, and MCI CHP 20P were purchased from Sigma-Aldrich Co. Octadecylsilica (ODS) gel (400–500 mesh) was obtained from Waters (New York, NY, USA). The activities were evaluated using the following reagents and enzymes: dimethyl sulfoxide (DMSO), bovine serum albumin (BSA), d-(−)-fructose, d-(+)-glucose, aminoguanidine hydrochloride, sodium azide, dl-glyceraldehyde, β-NADPH, 3,3′-tetramethyleneglutaric acid, ammonium sulfate, potassium phosphate buffer, Trizma-base, hydrochloric acid, porcine pancreatic lipase, *p*-nitrophenyl butyrate (*p*-NPB), orlistat, sodium phosphate buffer, α-glucosidase from *Saccharomyces cerevisiae*, acarbose, and 4-nitrophenyl α-d-glucopyranoside (*p*-NPG), all purchased from Sigma-Aldrich Co.

### 3.2. Plant Material

Samples of SCS for isolation were obtained from Yesan-gun, Chungcheongnam-do, Korea. Six samples of SCS for content analysis were collected from forests throughout different regions of Korea in August and botanically identified for content analysis. The stems were stored at 4 °C prior to extraction. Ten compounds: protocatechuic acid (**1**), 5-*O*-caffeoylquinic acid (**2**), 3-*O*-caffeoylquinic acid (**3**), 4-*O*-caffeoylquinic acid (**4**), kaempferol 3-*O*-β-d-glucopyranosyl-7-*O*-α-l-rhamnopyranoside (**5**), quercitrin (**6**), afzelin (**7**), *trans*-resveratrol (**8**), helonioside A (**9**), isoscutellarein 8-*O*-rhamnoside (**10**) were isolated as described in Section 3.3.

### 3.3. Extraction, Fractionation, and Isolation (Scheme 1)

**Scheme 1:**
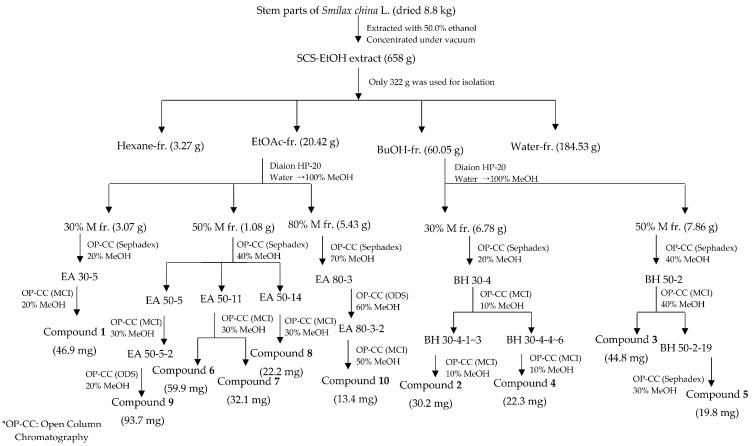
Extraction and isolation scheme of compounds **1**–**10** from stems of *Smilax china* L.

Fresh SCS samples (8800 g) were left to dry in the shade. Then, the dried stems were crushed and extracted using 50% ethanol (EtOH, 30 L ° 3 times at 25°C for 4 days). The 50% EtOH extract was concentrated under vacuum at 50 °C using a rotary evaporator, yielding 658 g of a sticky brown residue, of which only 322 g was used for product isolation. This aliquot was suspended in water and then successively partitioned with hexane (3 L ° 3), ethyl acetate (3 L ° 3) and *n*-butanol (6 L ° 3) to give 3.27 g, 20.42 g and 60.05 g of the corresponding extract, after solvent removal, in addition to a water-soluble fraction (184.53 g). Among these four fractions, the ethyl acetate fraction (EAF) and *n*-butanol fraction (NBF showed) manifested the most potent activities in the four antidiabetic model assays. Accordingly, repeated open column chromatography of both active fractions was performed (Scheme 1), yielding 10 compounds. To remove sugar, EAF and NBF were loaded onto a Diaion HP-20 column and eluted with the water, and then separated with 30%, 50%, 80%, and 100% methanol before being chromatographed on another gel column.

### 3.4. Component Identification

#### 3.4.1. NMR

Samples were dissolved in deuterated dimethyl sulfoxide (DMSO-*d*_6_), unless stated otherwise, and analyzed via 1D NMR (^1^H-NMR at 600 MHz, ^13^C-NMR at 150 MHz) and 2D NMR (^1^H-^13^C HMBC) spectroscopy using a VNS NMR 600 MHz spectrometer (Varian, Palo Alto, CA, USA). Chemical shifts are expressed as parts per million (ppm) on the δ scale.

#### 3.4.2. UHPLC-ESI/LTQ-Orbitrap-HRMS

Molecular weights of the isolated compounds were confirmed via ultrahigh performance liquid chromatography and high-resolution mass spectrometry (UHPLC-HRMS) coupled with electrospray ionization hybrid linear trap-quadruple-Orbitrap MS (ESI/LTQ-Orbitrap) using an Ultimate 3000 rapid separation liquid chromatography (RSLC) system (Thermo, Darmstadt, Germany). Samples were dissolved in 10% methanol. Mass analyses were performed in the both the positive and negative modes (polarity switching). The chromatographic separation was performed on a Hypersil GOLD C18 column (2.1 × 50 mm, 1.9 μm, Thermo) using a Nexera X2 system (Shimadzu, Kyoto, Japan). The mobile phase consisted of solvent A (0.1% aqueous formic acid) and solvent B (0.1% formic acid in acetonitrile). The gradient conditions were 0–18 min, solvent B 0%–50%; 18–19 min, solvent B 50%–100%. The flow rate was adjusted to 0.3 mL/min, and the column and sampler temperatures were maintained at 30 °C and 15 °C, respectively. The injection volume was 5.0 µL for the standard solution and 2.0 µL for the extract solution. UV wavelength was not used. The optimal conditions of the analysis were as follows: capillary temperature, 360 °C; heater temperature, 300 °C; sheath gas flow rate, 45 L/h; aux gas flow rate, 10 L/h; spray capillary voltage, 3.0 kV; S-lens RF level, 50.0 V; full MS resolution, 35,000 (FWHM @ *m*/*z* 200); full MS AGC target, 3e^6^; and full MS maximum IT, 200 ms. In the UHPLC-ESI/LTQ-Orbitrap-HRMS, negative mode was applied for all compounds except for compound **8**. The *m/z* data along with the retention time of each compound are provided in Table 5. All observed mass value accuracies are credible within the range of 5 ppm.

### 3.5. Structure Elucidation

**Figure 3:**
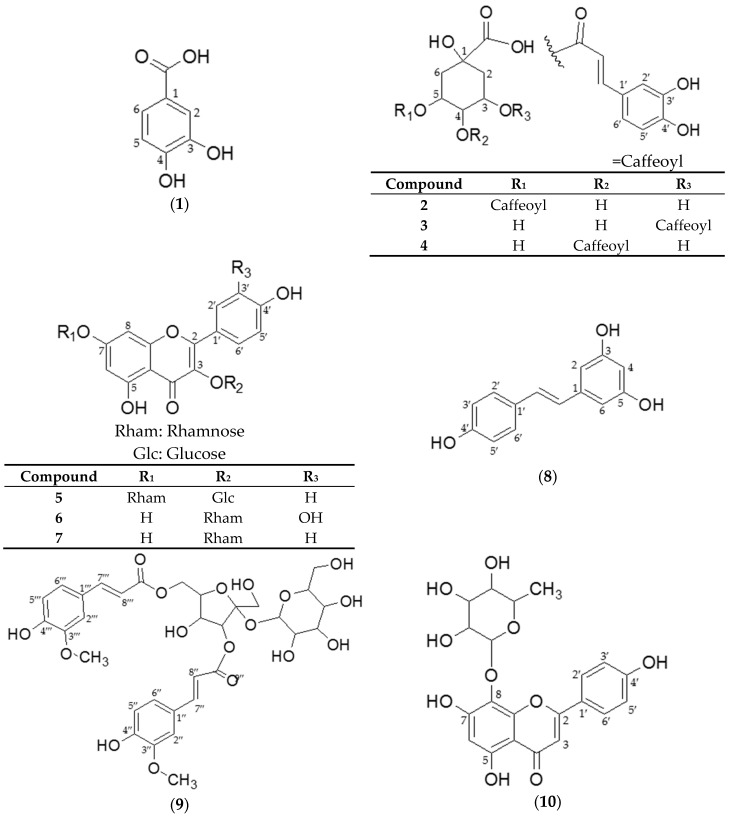
Chemical structures of compounds **1**–**10** isolated from *Smilax china* L. stem.

The structures of compounds **1**–**10** are shown in Figure 3.

*Protocatechuic acid* (**1**): Dark brown needle-shaped crystals; ^1^H-NMR: δ 7.34 (1H, s), 7.28 (1H, d, *J* = 6.6 Hz, H-6), 6.74 (1H, d, *J* = 7.5 Hz, H-5); ^13^C-NMR: δ 173.4 (C-7), 149.6 (C-4), 144.8 (C-3), 121.80 (C-1,6), 116.7 (C-2), 115.07 (C-5); (ESI) *m*/*z* = 153.02 [M − H]^−^.

*5-O-Caffeoylquinic acid* (**2**): Amorphous green powder; ^1^H-NMR (D_2_O in DMSO-*d*_6_): δ 7.47 (1H, d, *J* = 15.7 Hz, H-7′), 7.02 (1H, s, H-2′), 6.94 (1H, d, *J* = 7.6 Hz, H-6′), 6.78 (1H, d, *J* = 8.1 Hz, H-5′), 6.18 (1H, d, *J* = 15.8 Hz, H-8′), 5.21 (1H, m, H-5), 3.93 (1H, dd, *J* = 7.26, 2.5 Hz, H-3), 3.55 (1H, dd, *J* = 3.84, 1.02 Hz, H-4), 2.02 (1H, dd, *J* = 12.99, 4.68 Hz, H-6ax), 1.91 (1H, dd, *J* = 11.7, 3.54 Hz, H-6eq), 1.83 (1H, dd, *J* = 14.49, 7.02 Hz, H-2eq); ^13^C-NMR: δ 176.1 (C-7), 166.1(C-9′), 148.2 (C-4′), 145.6 (C-7′), 144.4 (C-3′), 125.7 (C-1′), 121.1 (C-6′), 115.8 (C-5′), 115.1 (C-2′), 114.6 (C-8′), 72.9 (C-1), 71.2 (C-5), 71.0 (C-4), 67.3 (C-3), 35.1 (C-2), 34.9 (C-6); (ESI) *m*/*z* = 354.31 [M − H]^−^.

*3-O-Caffeoylquinic acid* (**3**): Amorphous white powder; ^1^H-NMR (D_2_O in DMSO-*d*_6_): δ 7.38 (1H, d, *J* = 15.8 Hz, H-7′), 7.02 (1H, d, *J* = 1.9 Hz, H-2′), 6.97 (1H, dd, *J* = 2.0, 8.3 Hz, H-6′), 6.76 (1H, d, *J* = 8.2 Hz, H-5′), 6.10 (1H, d, *J* = 15.9 Hz, H-8′), 5.01 (1H, dd, *J* = 5.5, 11.5 Hz, H-3), 3.88 (1H, ddd, *J* = 6.15, 6.03, 3.24 Hz, H-5), 3.54 (1H, dd, *J* = 2.8, 5.6 Hz, H-4), 2.10 (2H, ddd, *J* = 15.9, 4.38, 3.78 Hz, H-2-eq, H-6eq), 1.93 (1H, dd, *J* = 3.2, 13.5 Hz, H-2ax), 1.76 (1H, dd, *J* = 12.6, 9.54 Hz, H-6ax); ^13^C-NMR: δ 173.6 (C-7), 165.4 (C-9′), 148.5 (C-4′), 145.6 (C-7′), 145.1 (C-3′), 125.4 (C-1′), 121.3 (C-6′), 115.9 (C-5′), 114.6 (C-2′), 113.9 (C-8′), 73.0 (C-1), 71.0 (C-5), 69.3 (C-4), 66.9 (C-3), 37.3 (C-2), 35.1 (C-6); (ESI) *m*/*z* = 354.31 [M − H]^−^.

*4-O-Caffeoylquinic acid* (**4**): Amorphous green powder; ^1^H-NMR (D_2_O in DMSO-*d*_6_): δ7.48 (1H, d, *J* = 15.9 Hz, H-7′), 7.04 (1H, d, *J* = 2.04 Hz, H-2′), 6.98 (1H, dd, *J* = 2.0, 8.3 Hz, H-6′), 6.77 (1H, d, *J* = 8.2 Hz, H-5′), 6.26 (1H, d, *J* = 15.9 Hz, H-8′), 4.69 (1H, dd, *J* = 6.93, 2.16 Hz, H-4), 4.10 (1H, dd, *J* = 7.0, 2.2 Hz, H-3), 3.88 (1H, br q, *J* = 2.6 Hz, H-5), 1.86 (2H, m, H-2ax, H-6eq), 1.78 (1H, dd, *J* = 11.49, 5.76 Hz, H-2eq), 1.22 (1H, m, H-2α, H-6ax); ^13^C-NMR: δ175.7 (C-7), 166.4(C-9′), 148.2 (C-4′), 145.5 (C-7′), 144.9 (C-3′), 125.7 (C-1′), 121.4 (C-6′), 115.8 (C-5′), 114.8 (C-2′), 114.6 (C-8′), 76.6 (C-1), 69.7 (C-5), 65.9 (C-4), 64.7 (C-3), 38.9 (C-2), 36.2 (C-6); (ESI) *m*/*z* = 354.31 [M − H]^−^.

*Kaempferol 3-O-β-d-glucopyranosyl-7-O-α-l-rhamnopyranoside* (**5**): Amorphous yellow powder; ^1^H-NMR: δ 8.07 (2H, d, *J* = 8.1, H-2′,6′), 6.89 (2H, d, *J* = 8.1, H-3′,5′), 6.82 (1H, s, H-8), 6.44 (1H, s, H-6), 5.54 (1H, s, H-1′′-glc), 5.47 (1H, d, *J* = 7.3, H-1′′′-rham), 3.17-4.02 (10H, m, H-2′′, H-3′′, H-4′′, H-5′′-rham, H-2′′′, H-3′′′, H-4′′′, H-5′′′, H-6a′′′, H-6b′′′-glc), 1.11 (3H, d, *J* = 5.7, H-6′′′); ^13^C-NMR: δ 177.7 (C-4), 161.6(C-7), 160.9 (C-4′), 160.2 (C-5), 156.8 (C-2), 156.0 (C-9), 133.5 (C-3), 131.0 (C-2′,6′), 120.7 (C-1′), 115.2 (C-3′,5′), 105.7 (C-10), 100.8 (C-1′′′-glc), 99.4 (C-1′′-rham), 77.6 (C-5′′′), 76.4 (C-3′′′), 74.2 (C-2′′′), 71.6 (C-4′′), 70.27 (C-4′′′), 70.09 (C-3′′), 69.93 (C-2′′), 69.8 (C-5′′), 60.9 (C-6′′′), 17.9 (C-6′′); (ESI) *m*/*z* = 594.52 [M − H]^−^.

*Quercetin 3-O-α-l-rhamnoside* (*quercitrin*, **6**): Amorphous yellow powder; ^1^H-NMR (D_2_O in DMSO-*d*_6_): δ 7.29 (1H, d, *J* = 2.1 Hz, H-2′), 7,24 (1H, dd, *J* = 2.1, 8,3 Hz, H-6′), 6.85 (1H, d, *J* = 8.3 Hz, H-5′), 6.36 (1H, s, H-8), 6.18 (1H, s, H-6), 5.24 (1H, d, *J* = 1.0 Hz, H-1′′-rham), 3.96 - 3.13 (4H, m, H-2′′, H-3′′, H-4′′, H-5′′), 0.80 (3H, d, *J* = 6.1 Hz, H-6′′); ^13^C-NMR: δ177.6 (C-4), 164.7 (C-7), 161.2 (C-5), 157.2 (C-2), 156.5 (C-9), 148.5 (C-4′), 145.2 (C-3′), 134.1 (C-3), 121.1 (C-6′), 120.7 (C-1′), 115.6 (C-5′), 115.4 (C-2′), 103.8 (C-10), 101.8 (C-1′′-rham), 98.8 (C-6), 93.6 (C-8), 71.2 (C-4′′), 70.6 (C-3′′), 70.3 (C-2′′), 70.0 (C-5′′), 17.5 (C-6′′); (ESI) *m*/*z* = 448.38 [M − H]^−^.

*Kaempferol 3-O-α-l-rhamnoside* (*afzelin*, **7**): Amorphous yellow powder; ^1^H-NMR (D_2_O in DMSO-*d*_6_): δ 7.73 (2H, d, *J* = 8.8 Hz, H-3′, H-5′), 6.90 (2H, d, *J* = 8.7 Hz, H-2′, H-6′), 6.37 (1H, s, H-8), 6.17 (IH, s, H-6), 5.28 (1H, s, H-1″-rham), 3.97–3.07 (4H, m, H-2″, H-3″, H-4″, H-5″), 0.78 (3H, d, *J* = 6.0 Hz, H-6); ^13^C-NMR: δ 177.5 (C-4), 165.0 (C-7), 161.2 (C-5), 160.0 (C-4′), 157.0 (C-2), 156.5 (C-9), 134.1 (C-3), 130.5 (C-2′, 6′), 120.5 (C-1′), 115.4 (C-3′,5′), 103.8 (C-10), 101.8 (C-1″-rham), 98.9 (C-6), 93.8 (C-8), 71.1 (C-4″), 70.6 (C-3″), 70.3 (C-2″), 70.1 (C-5″), 17.5 (C-6″); (ESI) *m*/*z* = 432.38 [M − H]^−^.

*3,5,4′-Trihydroxy-trans-stilbene* (*trans*-*resveratrol*, **8**): dark brown needle shaped crystals; ^1^H-NMR (D_2_O in DMSO-*d*_6_): δ 7.37 (2H, d, *J* = 8.6, H-2′,6′), 6.92 (1H, d, *J* = 16.4, H-b), 6.78 (1H, d, *J* = 16.4, H-a), 6.74 (1H, d, *J* = 8.6, H-3′,5′), 6.38 (2H, d, *J* = 2.04, H-2,6), 6.09 (1H, t, *J* = 2.1, H-4); ^13^C-NMR: δ 158.6 (C-3,5), 157.3 (C-4′), 139.7 (C-1), 128.5 (C-1′), 128.3 (C-b), 128.2 (C-2′, 6′), 125.9 (C-a), 115.8 (C-3′, 5′), 104.6 (C-2,6), 102.0 (C-4); (ESI) *m*/*z* = 229.24 [M + H]^+^.

*2-Feruloyl-O-α-d-glucopyranoyl-(1→2)-3,6-O-feruloyl-β-d-fructofuranoside* (*helonioside A*, **9**): Amorphous white powder; ^1^H-NMR (D_2_O in DMSO-*d*_6_): δ 3.46–4.50 (12H, m, H-4, H-2, H-1′b, H-1′a, H-3, H-6a, H-6b, H-5, H-5′, H-4′, H-6′b and H-6′a), 5.20 (1H, *J* = 3.42 Hz, H-1), 5.43 (d, 1H, d, *J* = 8.16 Hz, H-3′), *trans*-p-feruloyl units: R1: 3.80 (3H, -OCH_3_), 6.42 (d, 1H, *J* = 15.9 Hz, H-8′′), 6.80 (t, 1H, *J* = 8.1 Hz, H-5′′), 7.15 (d, 1H, *J* = 8.16 Hz, H-6′′), 7.32 (d, 1H, *J* = 7.7 Hz, H-2′′), 7.57 (d, 1H, *J* = 15.7 Hz, H-7′′), R2: 3.80 (s, 3H, -OCH_3_), 6.50 (d, 1H, *J* = 15.5 Hz, H-8′′′), 6.80 (t, 1H, *J* = 8.1 Hz, H-5′′′), 7.13 (d, 1H, *J* = 8.52 Hz, H-6′′′), 7.29 (d, 1H, *J* = 4.6 Hz, H-2′′′), 7.61 (d, 1H, d, *J* = 15.8 Hz, H-7′′′); ^13^C-NMR: δ 167.1 (C-9′′), 166.6 (C-9′′′), 149.7 (C-4′′), 148.0 (C-4′′′), 145.8 (C-3′′′), 145.5 (C-3′′), 145.3 (C-7′′′), 145.2 (C-7′′), 125.4 (C-1′′), 123.4 (C-1′′′), 123.2 (C-6′′), 123.0 (C-6′′′), 115.7 (C-5′′), 115.5 (C-5′′′), 114.1 (C-8′′), 114.0 (C-8′′′), 111.6 (C-2′′), 111.3 (C-2′′′), 104.3 (C-2′), 91.7 (C-1), 79.6 (C-5′), 76.9 (C-3′), 73.2 (C-4′,3), 73.0 (C-5), 72.8 (C-2), 71.5 (C-4), 65.6 (C-6′), 65.1 (C-1′), 63.0 (C-6), R_1_: 55.7 (-OCH_3_), R_2_: 55.7 (-OCH_3_); (ESI) *m*/*z* = 694.63 [M − H]^−^.

*Isoscutellarein 8-O-rhamnoside* (**10**): Amorphous yellow powder; ^1^H-NMR (D_2_O in DMSO-*d*_6_): δ 8.11 (2H, d, *J* = 7.3, H-2′,6′), 6.93 (2H, d, *J* = 8, H-3′,5′), 6.80 (1H, s, H-3), 6.40 (1H, s H-6), 5.53 (1H, s, H-1′′-rham), 3.17–3.85 (4H, m, H-rham), 1.13 (3H, d, *J* = 6, H-6′′); ^13^C-NMR: δ 181.4 (C-4), 165.9 (C-2), 160.6 (C-4′), 159.1 (C-5), 155.8 (C-7), 139.7 (C-9), 132.0 (C-3), 129.6 (C-2′, 6′), 127.5 (C-8), 121.9 (C-1′), 115.5 (C-3′,5′), 109.7 (C-10), 106.1 (C-1′′′-rham), 98.4 (C-6), 71.6 (C-4″), 70.2 (C-3″), 70.1 (C-2″), 69.8 (C-5″), 18.0 (C-6″); (ESI) *m*/*z* = 432.38 [M − H]^−^.

### 3.6. Biological Evaluation

#### 3.6.1. AGEs Formation Inhibition Assay

In accordance with a reported protocol [64] with slight modifications, a reaction mixture of bovine serum albumin (10 mg/mL, Sigma Aldrich) in 50 mM phosphate buffer (pH 7.4) with 0.02% sodium azide was added to 0.4M fructose and glucose. The reaction mixture (270 μL) was added to 30 μL of sequentially diluted compounds or the positive control, AMG. Each sample of extract, hexane, water fractions, EAF, NBF, and compounds **1**–**10** were dissolved in DMSO for the AGEs assay at five different final concentrations (10–500 µg/mL for extracts and fractions or 10–500 µM for isolated constituents). After 48 h of incubation at 60 °C, 200 μL of the reaction products was taken to 96-well plates and its fluorescence was measured using a spectrofluorometric detector (FlexStation 3, HTRF^®^ Chemistry) at an excitation wavelength of 350 nm and an emission wavelength of 450 nm. DMSO was used as negative control instead of the sample. The IC_50_ values and 95% confidence intervals were obtained via regression analysis. The percentage of inhibition (%) was calculated by the following equation, where Ac stands for the fluorescence of the control and As stands for the fluorescence of the sample:

(1) $$\mathrm{Inhibition}(\%)=\frac{\mathrm{Ac}-\mathrm{As}}{\mathrm{Ac}}\times 100$$

#### 3.6.2. AR assay

All experimental protocols for animal care and use were approved by the local ethics board (South Korean Institute of Oriental Medicine Animal Care and Use Committee; Protocol number 16–1045, 11 November 2016). Animal husbandry and procedures were performed in accordance with institutional guidelines. Lenses (one lens per 0.5 mL sodium buffer) were removed from Sprague-Dawley rats (weight 250–280 g) and stored in a deep freezer at −80 °C until use. The rat lenses were homogenized and centrifuged at 10,000 rpm (4 °C, 20 min) and the supernatant was used as an enzyme source [65]. AR activity was spectrophotometrically calculated by measuring the decrease in β-NADPH absorption rate at 340 nm for a 4 min period at 25 °C in a 96-well plate using the same detector device used in the AGEs assay. The assay mixture (300 µL) contained 0.1 M potassium phosphate buffer (pH 7.0), 0.1 M sodium phosphate buffer (pH 6.2), 1.6 mM β-NADPH, and test samples with 0.025 M of dl-glyceraldehyde as substrate. All extract samples, including hexane, water, EA, and NB fractions, as well as compounds **1**–**10**, were dissolved in DMSO for the AR assay at five different final concentrations (10–100 µg/mL for extracts and fractions or 0.1–100 µM for isolated constituents). IC_50_ values were determined using the least-squares regression line of log concentration plotted against residual activity. TMG, a typical AR inhibitor, was used as the positive control. A negative control was fulfilled by using DMSO instead of the sample.

#### 3.6.3. α-Glucosidase Inhibition Assay

This enzyme inhibition assay was carried out by using a 96-well microplate reader and a spectrophotometer. The reaction volume (60 μL) contained 100 mM phosphate buffer (pH 6.8), 2.5 mM *p*-nitrophenyl α-d-glucopyranoside (*p*-NPG), and sample dissolved in 10% DMSO at five different final concentrations (5–100 µg/mL for extracts and fractions or 1–1000 µM for isolated constituents). A chromogenic substance was used for quantification purposes. The mixture solution was added to each well, then, 20 μL of 10 mM phosphate buffer (pH 6.8) containing 0.2 U/mL α-glucosidase was added. The plate was incubated at 37 °C for 15 min, then, 80 μL of 0.2 mol/L sodium carbonate solution was added to stop the reaction. Absorbance was measured at 405 nm immediately after reaction cessation by using a SUNRISE microplate reader (Tecan Austria GmbH, Grodig, Austria). The negative control contained the same reaction mixture with phosphate buffer substituted for the sample solution. Acarbose was dissolved in 10% DMSO and used as the positive control. The inhibition rate of α-glucosidase activity was calculated using the same equation (1) used for inhibition of AGEs formation, but absorbance was measured instead of fluorescence.

#### 3.6.4. Lipase Inhibition Assay

Lipase inhibition activity was evaluated according to a slightly modified method of de Camargo et al. [66]. The substrate used was *p*-nitrophenyl butyrate (*p*-NPB), which is converted to *p*-nitro-phenol in the presence of lipase, giving a yellow color that can be used to quantify the extent of the reaction. Type II porcine pancreatic lipase was used at a concentration of 5 mg/mL. The substrate (*p*-NPB) was prepared in DMSO to achieve a concentration of 5 mM. Tested samples, dissolved in 10% DMSO at five different final concentrations (0.05–5.0 mg/mL for extracts and fractions or 10–2000 µM for isolated constituents), were mixed with Tris–HCl buffer (pH 7.4), and enzyme solution, with a total reaction mixture volume of 840 μL. After incubation at 37 °C for 15 min, *p*-NPB was added, and the solution was further incubated at 37 °C for 25 min. Then, the mixture was cooled for 2 min, and 200 μL was transferred to a 96-well plate. Absorbance was measured using the SUNRISE microplate reader. Blanks, in which enzymes were replaced with Tris–HCl buffer, were prepared for background corrections. The negative control consisted of all solutions except for the samples, and the common lipase inhibitor, orlistat, was used as the positive control. The inhibition rate of lipase activity was also calculated using Equation (1).

### 3.7. Quantitative Analysis by HPLC

To analyze the four compounds, 3-*O*-caffeoylquinic acid, 4-*O*-caffeoylquinic acid, quercitrin and *trans*-resveratrol, from the SCS extract, reversed-phase HPLC was performed using a Waters 600 pump (Waters, Milford, MA, USA) equipped with a Waters 996 photodiode array detector (Waters) for detection, a Waters 717 plus (Waters) autosampler, a YL CTS30 (Younglin, Anyang, Korea) column oven, and an ERC-3415 (ERC Inc., Saitama, Japan) degasser. Samples were separated using a RPAQUEOUS C30 column (Develosil, Nomura Chemical, Seto, Japan, 4.6 × 250 mm, 5 μm inner diameter) at 30 °C. Solvent A (water/phosphoric acid = 98:2, *v*/*v*) and solvent B (acetonitrile) were used as gradient linear mobile phases (0–10 min, 10%–20% B; 10–20 min, 20%–30% B; 20–60 min, 30%–35% B; 60–80 min 35%–80% B) at a flow rate of 1.0 mL/min. The injection volume was 10 µL and absorbance wavelength was 320 nm. For preparation of extract stock solutions, plant powders were sonicated with 70% MeOH for 90 min and dried under vacuum by using a rotary evaporator at 50°C. Then, they were dissolved in 70% MeOH to a concentration of 10,000 ppm. Standard compound stock solutions were dissolved in 100% MeOH. All analyzed solutions were strained using a 0.45-µm PVDF membrane filter prior to injection. The standard calibration curve was constructed using five concentrations. The linear relationship between peak area and concentration is described in (Table 6). The concentrations of the four major compounds were calculated using the regression equations based on the calibration curves.

### 3.8. Statistical Analysis

Statistical significance was analyzed via a one-way ANOVA and Student’s *t*-test to determine the differences between the positive control and treatment groups, established at *p* < 0.05. Data was presented as mean ± standard deviation (SD).

## 4. Conclusions

In this study, with the aim of searching for novel therapies for diabetic complications, we evaluated the capacities of four stem extracts from the family Smilacaceae to inhibit AGEs formation and AR activity. Since we identified that SCS extracts exhibited the most potent inhibitory capacities, we isolated the 10 major bioactive compounds of SCS and characterized their structures. All solvent fractions and isolated compounds were investigated for their antidiabetic potential, with most of the isolated compounds showing an inhibitory potency, with compounds **3**, **4**, **6**, and **8** possessing the highest inhibition capabilities. Therefore, we investigated the relation between the inhibition potency and the quantities of these four compounds in extracts from six SCS samples collected from different provinces of Korea. Samples from Gyeongsangnam-do Province possessed both the strongest inhibitory activity and the highest levels of the aforementioned four compounds. Isolated compounds from SCS also demonstrated powerful inhibitory activities against α-glucosidase and lipase enzymes. Consequently, SCS extracts could possess inhibitory effects against oxidative stress-related diabetic complications. 3-*O*-Caffeoylquinic acid, 4-*O*-caffeoylquinic acid, quercitrin, and *trans*-resveratrol obviously played vital roles in mediating these effects. Additionally, we also figured out that when using SCS for medicinal purposes, it is best to collect or cultivate the plant from Gyeongsangnam-do Province of Korea. Further studies are required to evaluate if the activity is sufficient to be used in clinical applications.

## Figures and Tables

**Table 1:** Inhibition of AGEs formation and AR activity by 70% MeOH stem extracts of selected members of the family Smilacaceae.

Test Materials	AGEs	Aldose Reductase
	IC_50_ (μg/mL)	IC_50_ (μg/mL)
*Smilax china* L.	145 ± 8 **	37.0 ± 4.6 **
*Smilax sieboldii*	366 ± 29 ***	80.8 ± 6.3 **
*Smilax riparia* var. ussuriensis	413 ± 17 ***	41.6 ± 1.8 **
*Smilax ferox* Wall. ex Kunth	186 ± 11 **	37.0 ± 1.7 **
AMG ^a^	54.8 ± 2.1 ***	-
TMG ^b^	-	1.30 ± 0.10 ***

^a^ Used as positive control in AGEs formation inhibitory assay. ^b^ Used as positive control in AR activity inhibitory assay. ** *p* < 0.01 compared with the positive control group. *** *p* < 0.001 compared with the positive control group. The 50% inhibitory concentration is expressed as the mean ± SD (*n* = 3).

**Table 2:** Inhibitory effect of SCS and solvent fractions on AGEs formation, AR, of α-glucosidase and lipase activities.

Test Materials	AGEs	Aldose Reductase	α-Glucosidase	Lipase
	IC_50_ (μg/mL)	IC_50_ (μg/mL)	IC_50_ (μg/mL)	IC_50_ (mg/mL)
SCS extract	145 ± 8 **	37.0 ± 4.6 **	51.7 ± 2.1 **	2.75 ± 0.22 **
Hexane fraction	ND	42.1 ± 1.1 **	41.6 ± 4.5 **	0.10 ± 0.02 *
EAF	40.5 ± 4.7 *	10.4 ± 0.3 **	9.48 ± 0.37 ***	0.14 ± 0.01 **
NBF	140 ± 18 *	39.0 ± 3.6 ***	33.8 ± 2.4	0.44 ± 0.03 **
Water fraction	410 ± 16 ***	ND	56.7 ± 1.1 *	4.12 ± 0.25 **
AMG ^a^	54.8 ± 2.1 ***	-	-	-
TMG ^b^	-	1.30 ± 0.10 ***	-	-
Acarbose ^c^	-	-	63.5 ± 1.9 ***	-
Orlistat ^d^	-	-	-	0.001 *

^a^ Used as positive control in AGEs formation inhibitory assay; ^b^ Used as positive control in AR activity inhibitory assay; ^c^ Used as positive control in α-glucosidase inhibitory assay; ^d^ Used as positive control in lipase inhibitory assay; * *p* < 0.05 compared with the positive control group; ** *p* < 0.01 compared with the positive control group; *** *p* < 0.001 compared with the positive control group; ND: no detection within test concentration; The 50% inhibitory concentration is expressed as the mean ± SD (*n* = 3).

**Table 3:** Inhibitory effect of SCS-isolated compounds **3**. Inhibition of AGEs formation, AR, α-glucosidase and lipase activities by isolated compounds from SCS.

Test Materials	AGEs	Aldose Reductase	α-Glucosidase	Lipase
	IC_50_ (µM)	IC_50_ (µM)	IC_50_ (µM)	IC_50_ (µM)
Protocatechuic acid (**1**)	418 ± 10 *	ND	176 ± 7	22.9 ± 0.8 ***
5-*O*-Caffeoylquinic acid (**2**)	215 ± 18 ***	87.5 ± 1.6 ***	389 ± 14	474 ± 35 **
3-*O*-Caffeoylquinic acid (**3**)	80.5 ± 5.5 **	0.60 ± 0.03 **	608 ± 5 ***	>1000
4-*O*-Caffeoylquinic acid (**4**)	74.1 ± 3.1 **	20.1 ± 0.1 ***	736 ± 15	>1000
Kaempferol 3-*O*-β-d-glucopyranosyl-7-*O*-α-l-rhamnopyranoside (**5**)	193 ± 9 **	9.38 ± 1.46	609 ± 32 ***	>1000
Quercitrin (**6**)	58.0 ± 2.5 **	0.56 ± 0.14 **	135 ± 13 *	96.7 ± 7.0 **
Afzelin (**7**)	75.4 ± 3.8 **	87.9 ± 2.1 ***	31.7 ± 1.6 **	572 ± 102 *
*trans*-Resveratrol (**8**)	148 ± 14 ***	60.5 ± 16.8 *	5.97 ± 0.20 **	61.2 ± 11.7 *
Helonioside A (**9**)	227 ± 16 **	34.5 ± 0.1 ***	154 ± 4	>1000
Isoscutellarein-8-*O*-rhamnoside (**10**)	440 ± 17 *	17.0 ± 3.6	45.1 ± 2.6 **	101 ± 18 *
AMG ^a^	515 ± 33 ***	-	-	-
TMG ^b^	-	6.97 ± 0.56 ***	-	-
Acarbose ^c^	-	-	172 ± 17 **	-
Orlistat ^d^	-	-	-	1.40 ± 0.12 *

^a^ Used as positive control in AGEs formation inhibitory assay; ^b^ Used as positive control in AR activity inhibitory assay; ^c^ Used as positive control in α-glucosidase inhibitory assay; ^d^ Used as positive control in lipase inhibitory assay; * *p* < 0.05 compared with the positive control group; ** *p* < 0.01 compared with the positive control group; *** *p* < 0.001 compared with the positive control group; ND: no detection within test concentration; The 50% inhibitory concentration is expressed as the mean ± SD (*n* = 3).

**Table 4:** Contents of the four major compounds in SCS extract samples (µg/ext. g).

Sample No.	Contents (µg/ext. g)			
	Compound 3	Compound 4	Compound 6	Compound 8
A	18.3 ± 0.1	1.15 ± 0.03	0.64 ± 0.02	0.049 ± 0.003
B	14.3 ± 0.8	0.68 ± 0.01	0.40 ± 0.06	1.90 ± 0.10
C	8.00 ± 1.08	1.01 ± 0.01	0.30 ± 0.02	0.90 ± 0.03
D	6.12 ± 0.26	0.62 ± 0.01	0.19 ± 0.01	1.83 ± 0.12
E	5.96 ± 0.61	0.36 ± 0.01	0.014 ± 0.005	0.30 ± 0.03
F	1.79 ± 0.08	0.32 ± 0.01	0.041 ± 0.019	0.44 ± 0.03

Gyeongsangnam-do (**A**), Gangwon-do (**B**), Jeollanam-do (**C**), Jeollabuk-do (**D**), Chungcheongnam-do (**E**), and Gyeonggi-do (**F**).

**Table 5:** Identification of 10 compounds in SCS via UHPLC-ESI/LTQ-Orbitrap-HRMS analysis

Compound name	Rt (min)	Formula	Mass Mode	Theoretical Mass	Observed Mass	Mass Error (Da)	Mass Accuracy (ppm)
Protocatechuic acid (**1**)	3.44	C_7_H_6_O_4_	N	153.01824	153.01837	0.00013	0.8
5-*O*-Caffeoylquinic acid (**2**)	5.03	C_16_H_18_O_9_	N	353.08671	353.08804	0.00133	3.8
3-*O*-Caffeoylquinic acid (**3**)	6.93	C_16_H_18_O_9_	N	353.08671	353.08820	0.00149	4.2
4-*O*-Caffeoylquinic acid (**4**)	7.34	C_16_H_18_O_9_	N	353.08671	353.08838	0.00167	4.7
Kaempferol 3-*O*-β-d-glucopyranosyl-7-*O*-α-l-rhamnopyranoside (**5**)	9.71	C_27_H_30_O_15_	N	593.15010	593.15179	0.00169	2.8
Quercitrin (**6**)	10.81	C_21_H_20_O_11_	N	447.09219	447.09372	0.00153	3.4
Afzelin (**7**)	12.12	C_21_H_20_O_10_	N	431.09727	431.09848	0.00121	2.8
*trans*-Resveratrol (**8**)	11.65	C_14_H_12_O_3_	P	229.08592	229.08633	0.00041	1.8
Helonioside A (**9**)	12.06	C_32_H_38_O_17_	N	693.20253	693.20441	0.00188	2.7
Isoscutellarein-8-*O*-rhamnoside (**10**)	13.08	C_21_H_20_O_10_	N	431.09727	431.09845	0.00118	2.7

N: negative mode; P: positive mode; Rt: retention time.

**Table 6:** Linear relation between peak area and concentration (*n* = 3).

Compound Number	Rt (min)	Regression Equation	r^2^	Linear Range (μg/mL)	LOD (μg/mL)	LOQ (μg/mL)
**3**	15.479	*y* = (2.0 × 10^6^)*x* + 13710	1.0000	1 - 250	0.96	2.92
**4**	17.416	*y* = (2.0 × 10^6^)*x* − 16531	1.0000	1 - 500	0.80	2.42
**6**	38.645	*y* = (2.0 × 10^6^)*x* + 26229	0.9997	1 - 500	2.78	8.44
**8**	45.640	*y* = (7.0 × 10^6^)*x* + 26716	0.9998	1 - 500	0.77	2.33

In the regression equation *y* = a*x* + b, *x* refers to the concentration of the compound (μg/mL), *y* the peak area; r^2^: the correlation of the equation; Rt: retention time; LOD: limit of detection; LOQ: limit of quantification.
